# Motives for dish choices during home meal preparation: results from a large sample of the NutriNet-Santé study

**DOI:** 10.1186/s12966-015-0270-9

**Published:** 2015-09-30

**Authors:** Pauline Ducrot, Caroline Méjean, Benjamin Allès, Philippine Fassier, Serge Hercberg, Sandrine Péneau

**Affiliations:** Université Paris 13, Equipe de Recherche en Epidémiologie Nutritionnelle, Centre de Recherche en Epidémiologie et Statistiques, Inserm (U1153), Inra (U1125), Cnam, COMUE Sorbonne Paris Cité, F-93017 Bobigny, France; Département de Santé Publique, Hôpital Avicenne, F-93017 Bobigny Cedex, France; Equipe de Recherche en Epidémiologie Nutritionnelle (EREN), SMBH Université Paris 13, 74 rue Marcel Cachin, F-93017 Bobigny Cedex, France

**Keywords:** Dish choices, Home-meal preparation, Cooking practices, Constraints, Cross-sectional study

## Abstract

**Background:**

Although culinary practices have strongly evolved over time, few data are available on contemporary dish choices during meal preparation. We therefore sought to determine individual motives when choosing dishes to be prepared during weekdays and on weekends.

**Methods:**

The importance of 27 criteria related to dish choices was assessed in 53,025 participants in the NutriNet-Santé study. Dimensions of dish choice motives were investigated using exploratory factor analysis. Mean ratings of motives during weekdays and on weekends were compared using Student's *t*-test. Association between socio-demographic and cooking practice characteristics, and dish choice motives were evaluated using logistic regression models.

**Results:**

Five dimensions of dish choice motives emerged: *healthy diet* (explained variance: 48.3 %)*, constraints* (19.0 %)*, pleasure* (12.1 %)*, specific diets* (11.0 %) and *organization* (9.6 %)*.* The *healthy diet* factor was the most important on weekdays (mean rating 3.93) and weekends (3.90). *Pleasure* (3.61) had a higher score than *constraints* (3.54) on weekends (*p* < 0.0001) while the opposite was observed on weekdays (3.42 vs 3.77, respectively) (*p* < 0.0001). *Organization* was more important on weekdays (2.89) than on weekends (2.75) (*p* < 0.0001). Dish choice motives appeared to be significantly associated with socio-demographic and cooking practice characteristics.

**Conclusion:**

This study highlighted factors involved in dish choices in meal preparation on weekdays and weekends, as well as individual characteristics which determine motives for dish choices. From a public health perspective, these findings might help to develop appropriate strategies for promoting home meal preparation.

**Electronic supplementary material:**

The online version of this article (doi:10.1186/s12966-015-0270-9) contains supplementary material, which is available to authorized users.

## Introduction

Over the last decades, overweight and obesity have become major public health concerns in many countries [1]. During the same period, dietary practices have considerably evolved. Less time is spent on home cooking and food preparation due to changing lifestyle and, in particular, increasing time devoted to work and leisure [2–4]. Despite a decline in home cooking, foods consumed at home in 2007–2008 still represented about 69 % of total daily energy intake in the United States [5]. In response to lack of time, however, the type of food prepared at home has evolved towards more convenience and ready-prepared foods [6, 7], frequently high in calories, fat and sodium [8]. Consumption of convenience foods has been associated with lower diet quality in children and adolescents [9] and a higher prevalence of overweight in adults [10].

Benefits have been attributed to foods cooked at home. People who reported more frequent food preparation at home were more likely to meet dietary recommendations [11]. They also consumed less fat and more fruits, vegetables, whole-grain foods, fiber, calcium, folates and vitamin A [12, 13]. Moreover, time spent in food preparation was inversely related to BMI in women [14] and was associated with a higher quality diet and, in particular, greater vegetable consumption [15, 16].

In light of these data, encouraging home meal preparation may be a lever to improve diet quality and nutritional status. From a public health point of view, it is important to identify factors governing food choices for meal preparation since home-cooking (vs. eating out) but also the type of food that is cooked impact the dietary quality. To our knowledge, no data are available in the literature on motives for choosing dishes for home meal preparation. Only one small-scale qualitative study described motives for choosing between different meal solutions which include homemade meals, ready meals, take-out food and eating out [17]. In that study, sensory appeal, health-related benefits and meal context, as well as time and energy for food preparation, were shown to play an important role in meal choices. Other studies related to home meal preparation mainly focused on barriers to cooking, such as lack of time [4, 18, 19], parental employment [20–23] or poor cooking skills [10].

Thus, the aim of the present study was to investigate, in a large population, motives for choosing dishes for home meal preparation. Due to potential differences in cooking practices on weekdays and the weekend [24], our second objective was to compare motives in each of these contexts. Finally, our third objective was to evaluate how socio-demographic and cooking practice characteristics were related to dish choice motives.

## Subjects and methods

### Study population

NutriNet-Santé (https://www.etude-nutrinet-sante.fr) is an ongoing web-based prospective observational cohort study launched in France in May 2009 with a scheduled follow-up of 10 years. It aims to investigate the relationship between nutrition and chronic disease risk, as well as the determinants of dietary behavior and nutritional status. The study was implemented in the general French population (internet-using adult volunteers, age ≥ 18 years). The rationale, design and methodology of the study have been fully described elsewhere [25]. In brief, to be included in the study, participants had to complete a baseline set of self-administered web-based questionnaires assessing dietary intake (at least two 24 h records), physical activity, anthropometric characteristics, lifestyle, socioeconomic conditions and health status. As part of the follow-up, participants are requested to complete the same set of questionnaires every year. Moreover, each month, participants are invited by e-mail to fill in optional questionnaires related to dietary intakes, determinants of eating behaviors, nutritional and health status. This study is conducted in accordance with the Declaration of Helsinki, and all procedures were approved by the Institutional Review Board of the French Institute for Health and Medical Research (IRB Inserm n°0000388FWA00005831) and the Commission Nationale de l’Informatique et des Libertés (CNIL n°908450 and n°909216). All participants provided informed consent with an electronic signature. This study is registered in EudraCT (n°2013–000929–31).

### Data collection

#### Dish choice questionnaire

Data concerning dish choices were collected in September 2013 via an optional questionnaire, available for six months on the web platform (Additional file 1). Information as to whether the participant was involved in the choice of dishes was collected (never, sometimes, often, always). The questionnaire assessing determinants of dish choices was generated based on existing literature and the expertise of nutritionists, epidemiologists, sociologists and sensory specialists. The questionnaire included 27 items on dish choice motives, including commonly recognized factors such as preferences, eating habits, cooking practices, health, constraints related to time and food availability. Participants were asked the following question: “When choosing the dishes you are going to cook, how important are the following criteria?”. The responses were rated on a 5-point Likert scale ranging from 1 (not important at all) to 5 (very important), with each point on the scale represented by a word anchor. Because cooking practices differ on weekdays compared to weekends [24], information about dish choice motives was collected for weekdays and weekends separately.

In addition, general information on cooking practices was self-estimated by participants for both weekdays and weekends i.e., time spent in meal preparation (< 15 min, 15–30 min, 30–45 min, > 45 min), cooking skills (low, medium, high) and cooking enjoyment (yes, no).

#### Socio-demographic and economic data

At baseline and each year thereafter, participants are requested to specify socio-demographic and economic data, including age, gender, presence of children, education level (up to secondary, some college or university) and income. The monthly household income is calculated per household consumer unit (CU). One CU is attributed to the first adult in the household, 0.5 CU - for other persons aged 14 or older, and 0.3 CU - for children under 14 [26]. The following categories were used < 1,200 €/CU, 1,200–1,800 €/CU, 1,800–2,700 €/CU, > 2,700 €/CU). For each participant, we used socio-demographic data collected closest to the date at which the questionnaire was filled in.

### Statistical analyses

We performed analyses on participants included in the NutriNet-Santé cohort study who completed the questionnaire on dish choice motives and who declared being involved in dish choices. Participants who were “never” involved in dish choices were excluded from analyses. Chi-square and non-parametric Wilcoxon’s rank-sum tests were used to compare included and excluded subjects, as appropriate.

Exploratory factor analysis was performed to identify dimensions of dish choice motives. Before processing the analysis, adequacy of the items’ common variance for factor analysis was examined using the Kaiser-Meyer-Olkin (KMO) test [27]. Since items are represented by ordinal variables, we used the unweighted least squares estimation method based on polychoric correlations [28]. Since the factors were expected to be correlated, oblique rotation (Promax option in SAS) was applied [27]. The number of “meaningful” factors to be retained was determined using: 1- the Kaiser criterion (only factors with an eigenvalue greater than 1.00 are retained) [29], 2- the scree test (factors that appear before the break are assumed to be meaningful) [30], 3- the proportion of variance accounted for (only factors that account for more than 5 or 10 % of the variance are retained) [29] and 4- the interpretability criteria (interpretation of factors retained must make sense) [29]. To interpret the rotated factor pattern, an item was considered to load onto a given factor if factor loading was higher than to or equal to 0.40 for that factor and less than 0.40 for the other factors [28]. Items having non-negligible loading (> 0.30) for several factors were removed from further analysis. To access the internal consistency of the factors, ordinal alpha coefficients were calculated. Although based on polychoric correlations, these coefficients are conceptually equivalent to Cronbach’s alpha [31]. Thus, their interpretation is similar, i.e. reliability is considered acceptable if the coefficient exceeds the threshold of 0.60–0.70 [32].

Scores on each of the factors drawn in exploratory factor analysis were computed by averaging unweighted ratings for individual items. Scores could therefore range from 1 to 5. Student's t-tests were performed to compare average ratings for each factor on weekdays and on weekends.

Finally, for each factor, logistic regression models were used to evaluate the association between socio-demographic and cooking practice characteristics and dish choice motives. The modeled probability was a motive rated as important (i.e. average score ≥ 4).

All tests of significance were two-sided, and a *P* value < 0.05 was considered significant. Statistical analyses were performed using SAS software (version 9.3; SAS Institute Inc.).

## Results

### Characteristics of the sample

Among the 150,725 subjects included in the NutriNet-Santé study in September 2013, 53,025 persons (35.2 %) completed the optional questionnaire. Within this sample, 1,379 declared that they were never involved in home dish choices and were therefore excluded, leaving 51,646 subjects for the present analysis. A total of 50,915 individuals completed the questionnaire for weekdays and 51,043 for weekends. Characteristics of the studied population are presented in Table 1. Compared with excluded subjects, included subjects were more often women, younger, had a higher education level, lower income, and were more likely to have children living in their household. As regards cooking practices, included subjects spent more time in meal preparation, had better cooking skills and enjoyed cooking more than excluded subjects.

**Table 1:** Socio-demographic, economic and cooking practice characteristics of included (*N* =51,646) and excluded (*N* = 1,379) participants (NutriNet-Santé study, 2013)

	Included	Excluded			
		Never involved in dish choices (not analyzed)	*p* ^*^	NutriNet-Santé cohort (not enrolled + not analyzed)	*p* ^*^
	(*N* = 51,646)	(*N* = 1,379)		(*N* = 99,079)	
	%	%		%	
Sex					
Women	79.0	15.4	< 0.0001	77.2	< 0.0001
Men	21.0	84.6		22.8	
Age					
18–30	10.9	4.1	< 0.0001	21.9	< 0.0001
30–50	35.9	13.2		45.6	
50–65	35.8	30.8		23.9	
> 65	17.4	51.9		8.6	
Education level					
Up to secondary	31.3	47.9	< 0.0001	38.4	< 0.0001
Some college	28.5	19.9		28.5	
University	34.4	26.5		29.1	
Missing data	5.7	5.7		4.0	
Monthly income per household unit (€/CU)^a^					
< 1,200	14.4	10.5	< 0.0001	22.7	< 0.0001
1,200–1,800	23.7	23.3		25.9	
1,800–2,700	24.7	24.8		21.1	
≥ 2,700	26.3	31.3		18.7	
Missing data	10.9	10.1		11.6	
Presence of children					
Yes	29.6	15.2	< 0.0001	38.7	< 0.0001
No	70.4	84.7		61.3	
Missing data	0	0.1		0	
Cooking skills					
Low	12.0	84.8	< 0.0001		
Medium	37.7	9.6			
High	50.3	5.5			
Time spent in meal preparation					
Weekdays					
< 15 min	12.3	80.6	< 0.0001		
15–30 min	42.5	11.0			
30–45 min	27.8	4.2			
≥ 45 min	17.4	4.1			
Weekends					
< 15 min	7.4	82.3	< 0.0001		
15–30 min	21.7	6.4			
30–45 min	30.7	5.0			
≥ 45 min	40.9	6.3			
Cooking enjoyment					
Weekdays					
Yes	68.4	14.1	< 0.0001		
No	31.6	85.9			
Weekends					
Yes	80.9	16.8	< 0.0001		
No	19.1	83.2			

*P-values based on non-parametric Wilcoxon test or chi-squared test

^a^CU: Household Consumer Units. One CU is attributed for the first adult in the household, 0.5 for other persons aged 14 or older and 0.3 for children under 14

Compared with the overall NutriNet-Santé population, participants of the present study were more often women, older, had a higher educational level, higher income, and were more likely to have no children in their household.

### Motives for dish choices

Mean ratings of the 27 motives for dish choices are presented in Table 2. Data are presented by order of importance on weekdays. Whatever the context, the items “To cook with seasonal products” and “My preferences and/or that of my relatives” were, respectively, ranked first and second as motives when choosing dishes to cook. On weekdays, “The ingredients I have at my disposal” and “The nutritional balance of the dish” were rated next in order of importance. In turn, on weekends, “What I and/or my relatives want to eat” was next in order of importance.

**Table 2:** Mean ratings of the 27 motives for dish choices (N = 51,646, NutriNet-Santé study, 2013)

	Mean ratings^a^	
When choosing the dishes you plan to cook, how important are the following criteria?	Weekdays	Weekends
Use of seasonal products	4.24	4.27
My preferences and/or those of my relatives	4.19	4.26
Ingredients at my disposal	4.14	3.94
Nutritional balance of the dish	4.00	3.93
Nutritional balance of the meal	3.94	3.91
What I and/or my relatives want to eat	3.90	4.07
Time available for cooking	3.87	3.43
My eating habits and/or those of my relatives	3.82	3.74
Leftovers in my refrigerator/freezer	3.71	3.38
My state of fatigue	3.68	3.44
My state of hunger and/or that of my relatives	3.67	3.61
What I and/or my relatives ate during the previous days	3.64	3.64
The association with other dishes in terms of taste	3.61	3.73
Number of persons eating at home	3.61	3.73
My cooking skills	3.57	3.44
Price of ingredients	3.57	3.44
My health status and/or those of my relatives	3.45	3.44
Cooking equipment I possess	3.43	3.44
The dish can be adapted to please all guests	3.40	3.51
The dish can be prepared beforehand	3.13	2.84
My eventual diet to lose weight and/or that of my relatives	3.04	2.97
The dish can be prepared in large quantities	2.98	2.84
Recipes I come across	2.77	3.03
Originality of the dish	2.67	3.04
The dish is easy to eat	2.66	2.44
What I planned to eat (meal planning)	2.55	2.57
My personal convictions and/or that of my relatives	1.94	2.00

^a^Responses were rated on a 5-point Likert scale ranging from 1 (not at all important) to 5 (very important)

### Factor analysis

Given the KMO measure (0.83), the data presented adequate common variance enabling exploratory factor analysis. All criteria used to determine the number of meaningful factors converged into a five-factor solution. Four items included in the analysis presented low loading (< 0.40) for all factors and were thus removed from further analysis, i.e. “The dish is easy to eat”, “The number of persons eating at home”, “The cooking equipment I have”, “The price of ingredients”. In turn, one item had cross-loadings > 0.40 for two factors, i.e. “The association with other dishes in terms of taste”, and was therefore excluded. As a result, 22 items were retained among the initial 27 items.

Results of explanatory factor analysis are shown in Table 3. The first factor explained 48.3 % of the total variance and consisted of 5 items corresponding to healthy eating motives. The second factor accounted for 19.0 % of the total variance and included 6 items, all referring to constraints. The third factor accounted for 12.1 % of the total variance and comprised five items referring to pleasure. The fourth factor explained 11.0 % of the total variance and consisted of three items related to specific diets. Finally, the fifth factor accounted for 9.6 % of the total variance and included 3 items concerning meal organization.

**Table 3:** Explanatory factor analysis (factor loadings and internal consistency) of motives for dish choices (N = 51,646, NutriNet-Santé study, 2013)

When choosing the dishes you plan to cook, how important are the following criteria?	Standardized factor loading	Internal consistency
Factor 1: Healthy diet		0.75
Nutritional balance of the meal	0.87	
Nutritional balance of the dish	0.81	
Use of seasonal products	0.51	
My eating habits and/or that of my relatives	0.40	
What I and/or my relatives ate during the previous days	0.40	
Factor 2: Constraints		0.68
Ingredients at my disposal	0.61	
Leftovers in my refrigerator/freezer	0.53	
My state of fatigue	0.53	
Time available for cooking	0.51	
My hunger and/or that of my relatives	0.45	
My cooking skills	0.41	
Factor 3: Pleasure		0.66
What I and/or my relatives want to eat	0.62	
Originality of the dish	0.55	
My preferences and/or those of my relatives	0.54	
Recipes I come across	0.47	
The dish can be adapted to please all guests	0.39	
Factor 4: Specific diets		0.69
My health status and/or that of my relatives	0.75	
My eventual diet to lose weight and/or that of my relatives	0.64	
My personal convictions and/or that of my relatives	0.47	
Factor 5: Organization		0.64
The dish can be prepared beforehand	0.71	
The dish can be prepared in large quantities	0.51	
What I planned to eat (meal planning)	0.49	

To assess potential differences in dish choice motives between men and women, factor analyses were performed independently for each sex. Data indicated very similar results, apart from two items. For men, the item “What I and/or my relatives ate during the previous days” did not load into the *healthy diet* factor, whereas it did so for women and in global analyses. In contrast, the item “The dish is easy to eat” loaded in the *constraints* factor for men but not for women, nor in global analyses. Men had lower mean scores for all factors compared to women. However, overall, factor scores were ranked the same in men and women. Given the few differences between men and women, analyses were conducted on the whole sample.

Intercorrelations between factors were calculated. All correlation coefficients were significant but below 0.50. Thus, we can consider that there existed no multicollinearity in the present data [27] and that dimensions were distinct from one another.

### Comparison of weekdays with weekends

Table 4 shows the importance of each factor in the two contexts: weekdays and weekends. Among the five factors, three were considered as more important on weekdays (i.e. *healthy diet*, *constraint*s and *organization*), one was ranked as more important during the weekend (i.e. *pleasure*) and one showed no differences between the two contexts (i.e. *specific diets*).

**Table 4:** Mean ratings of the importance of each dish choice factor in both contexts (weekdays and weekends) (N = 51,646, NutriNet-Santé study, 2013)

	Healthy diet	Constraints	Pleasure	Specific diets	Organization
	*P* < 0.0001	*P* < 0.0001	*P* < 0.0001	*P* = 0.35	*P* < 0.0001
Weekdays	3.93	3.77	3.42	2.81	2.89
Weekends	3.90	3.54	3.61	2.80	2.75

Responses were rated on a 5-point Likert scale ranging from 1 (not at all important) to 5 (very important)

As regards ranking, the *healthy eating* dimension was the most important factor when choosing dishes on both weekdays and weekends, followed by *constraints *and *pleasure*. *Constraints* were more important than *pleasure* on weekdays, but the opposite was true on weekends. *Organization* and *specific diets* ranked last. In the case of weekdays, *organization* was more important than *specific diets* and the reverse was observed for weekends.

Differences between men and women were assessed. For weekdays, men and women indicated the same ranking as the overall group. For weekends, *specific diets* was the least important in men, rather than *organization* both in general ranking and in women. Moreover, for all factors and whatever the context, means score were lower in men than in women.

### Association between socio-demographic and cooking practice characteristics, and dish choice motives

Logistic regression analysis showing the associations between socio-demographic and cooking practice characteristics, and dish choice motives are presented in Table 5.

**Table 5:** Logistic regression model showing the association between socio-demographic and cooking practice characteristics, and dish choice motives (*N* = 49,537, NutriNet-Santé study, 2013)^a^

	Healthy diet				Constraints				Pleasure				Specific diets				Organization			
	OR	95 % CI		*p* ^b^	OR	95 % CI		*p* ^b^	OR	95 % CI		*p* ^b^	OR	95 % CI		*p* ^b^	OR	95 % CI		*p* ^b^
Sex																				
Men	1			< .0001	1			< .0001	1			< .0001	1			< .0001	1			< .0001
Women	2.40	2.29	2.52		2.76	2.61	2.91		1.65	1.54	1.77		2.20	2.03	2.39		1.87	1.73	2.03	
Age																				
18–30	1			< .0001	1			< .0001	1			< .0001	1			< .0001	1			< .0001
30–50	1.53	1.43	1.63		0.80	0.75	0.86		1.06	0.97	1.16		0.95	0.87	1.03		1.20	1.09	1.32	
50–65	2.10	1.96	2.24		0.46	0.43	0.49		1.25	1.15	1.37		0.70	0.65	0.76		1.00	0.91	1.10	
≥ 65	2.05	1.90	2.22		0.30	0.28	0.32		1.30	1.18	1.43		0.58	0.52	0.64		0.98	0.88	1.09	
Education																				
Up to Secondary	1			< .0001	1			< .0001	1			< .0001	1			< .0001	1			< .0001
Some college	1.20	1.15	1.26		1.07	1.02	1.12		0.82	0.77	0.87		0.86	0.81	0.92		0.91	0.86	0.97	
University	1.26	1.20	1.32		1.23	1.17	1.29		0.66	0.62	0.70		0.67	0.62	0.71		0.80	0.74	0.85	
Monthly income per household unit (€/CU)^c^																				
< 1,200	1			< .0001	1			< .0001	1			0.004	1			< .0001	1			0.023
1,200–1,800	0.96	0.94	1.07		0.96	0.90	1.02		0.96	0.88	1.03		0.78	0.72	0.85		1.05	0.96	1.14	
1,800–2,700	1.11	1.04	1.18		0.91	0.86	0.97		0.96	0.88	1.04		0.74	0.68	0.80		1.13	1.04	1.24	
≥ 2,700	1.13	1.05	1.20		0.84	0.79	0.90		1.01	0.93	1.09		0.62	0.57	0.68		1.04	0.95	1.14	
Missing data	1.08	1.00	1.16		0.93	0.86	1.00		1.11	1.02	1.22		0.91	0.83	1.00		1.11	1.01	1.23	
Presence of children in the household																				
No	1			0.48	1			0.011	1			< .0001	1			< .0001	1			0.36
Yes	0.98	0.94	1.03		1.07	1.02	1.12		0.77	0.72	0.83		0.69	0.65	0.74		0.97	0.90	1.04	
Cooking skills																				
Low	1			< .0001	1			0.024	1			< .0001	1			0.0012	1			< .0001
Medium	1.16	1.09	1.24		1.08	1.00	1.15		1.18	1.06	1.32		0.86	0.78	0.94		1.20	1.07	1.33	
High	1.66	1.54	1.78		1.02	0.95	1.10		1.87	1.66	2.09		0.92	0.83	1.02		1.38	1.24	1.55	
Time spent in meal preparation (*weekdays)*																				
0–15 min	1			< .0001	1			0.011	1			< .0001	1			< .0001	1			< .0001
15–30 min	1.28	1.20	1.37		1.02	0.96	1.09		1.09	0.99	1.21		1.04	0.94	1.14		1.06	0.96	1.18	
30–45	1.55	1.45	1.66		0.94	0.88	1.01		1.33	1.20	1.47		1.23	1.11	1.36		1.17	1.06	1.31	
≥ 45 min	1.73	1.60	1.87		0.97	0.90	1.05		1.54	1.38	1.72		1.28	1.15	1.43		1.43	1.28	1.60	
Cooking enjoyment *(weekdays)*																				
No	1			< .0001	1			< .0001	1			< .0001	1			< .0001	1			0.51
Yes	1.63	1.56	1.71		0.77	0.73	0.81		1.81	1.69	1.94		1.26	1.17	1.34		1.02	0.96	1.09	

^a^Model was adjusted for sex, age, education level, income, presence of children, cooking skills, time spent in meal preparation (weekdays) and cooking enjoyment (weekdays)

^b^The modeled probability was a motive ranked as important (i.e. average score ≥ 4)

^c^CU: Household Consumer Units. One CU is attributed for the first adult in the household, 0.5 for other persons aged 14 or older and 0.3 for children under 14

Individuals who gave importance to a *healthy diet* when choosing dishes were more likely to be women, to be older, to have a higher educational level and to have a higher income. They also declared greater cooking skills, spent more time in meal preparation and enjoyed cooking more.

Participants who felt that *constraints *were important were more likely to be women, younger, to have a lower income, to have children living in the household and were less likely to enjoy cooking.

Individuals who gave importance to *pleasure* were more likely to be women, to be older, to have a lower educational level and no child living in the household. They also declared greater cooking skills, spent more time in meal preparation and enjoyed cooking more.

Participants who reported importance for *specific diet* were more likely to be women, younger, to have lower educational level, lower income and no child living in the household. They also had lower cooking skills, spent more time in meal preparation and reported higher cooking enjoyment.

Finally, individuals who gave importance to *organization* were more likely to be women, to be 30–50 year old, to had lower educational level, to have a monthly income between 1,800 and 2,700 €. They also declared greater cooking skills and spent more time in meal preparation.

## Discussion

In the present study comprising a large sample of individuals, we describe for the first time motives for dish choices during home meal preparation. Based on results of explanatory factor analysis, we identified five dimensions underlying dish choices: *healthy diet, constraints, pleasure, specific diets* and *organization*. Comparison between weekdays and weekends revealed that *healthy diet, constraints* and *organization* were more important on weekdays, while *pleasure* was more important on weekends. Finally, dish choice motives appeared to be significantly associated with socio-demographic and cooking practice characteristics.

### Dimensions underlying dish choices

Our findings suggest that health is the most important criterion when choosing dishes for home cooking. In the literature, health has generally been identified as an important food choice motive [33–37]. In the present study, the *healthy diet* factor contained items related to nutrition and, in particular, nutritional balance and diet variety, consistent with the balanced diet definition of the Food and Agriculture Organization (FAO) and messages issuing from public health policies [38]. The main importance of this factor is in line with the French idea that home cooking is a means of staying healthy [39], which might be explained by the association of the French Mediterranean cooking patterns based on raw ingredients with healthiness [40, 41]. In addition, the *health diet* factor includes items concerning seasonal products. Over the last decades, French became more sensitive to additives in foodstuffs and attach an increasing importance in consuming natural products (e.g. contaminant free, additive free), which they consider as better for health [42, 43]. Consuming seasonal product has been reported as a growing trend in France but also in the rest of Europe [42, 44]. In line with this idea, studies on food choice motives suggested a strong association between health and natural content motives [34, 35, 37].

The *specific diets* factor consisted of items concerning diet practices related to health status, weight loss strategy or personal conviction (e.g. vegetarianism, religion). In contrast with the *healthy diet* factor that relates to overall eating behavior, this factor focuses on the practice of specific diets which only concerns a subgroup of population. This could explain its lesser importance compared to the *healthy diet* factor. However, the emergence of this factor in the explanatory factor analysis suggests that practices related to such diets are important criteria in dish choices. Indeed, such diets often require a lot of attention on the type of food that is prepared.

*Pleasure* also emerged as a significant factor in dish choices and included items on preferences, sociability and novelty. Studies that focused on food choices identified sensory appeal as an important motive in food selection [33–37]. In line with the literature, preferences of the person choosing the dish, but also of persons sharing the meal, were shown to be important. Indeed, cooking is often described as a social event [45, 46]: more pleasure is procured from cooking when others partake of the meal. If no time pressure exists, then cooking can even be described as a leisure-time activity [24] which is likely to favor creativity and originality. The emergence of this factor in our sample highlight the importance of meal conviviality that had previously been shown to be more important in French people, compared with other western cultures such as English [41] or German [47].

The *constraints* factor included items about time, cooking skills, food availability (ingredients and leftovers) and physiological condition (fatigue and hunger). In agreement with the literature, time pressure [18, 21–23] and cooking skills [10] have already been described as being major barriers to home meal preparation. Previous studies have shown that fatigue may lead to the use of quick and easy food solutions [21], generally of low nutritional quality. Hunger was also described as a modulator of food choices not only in terms of food quantities consumed, but also in the choice of products [48].

The *organization* factor included items focusing on meal planning. A number of studies suggest that meal planning may increase family dinner frequency, reduce the use of convenience foods [49] and increase consumption of fruits and vegetables [16]. Meal planning tools have therefore been proposed by nutritionists and public health programs so as to limit constraints related to home-meal preparation and to improve diet quality. In our study, this factor appeared to be of lesser importance. A potential hypothesis to explain this result might be that such practices require a lot of organization leading to a small proportion of individuals managing to maintain such practices over a long-term period. In particular, one previous study highlighted that women who experienced time pressure are less likely to plan meals [49]. Another explanation might be that, due to retail food availability [50], it is no longer necessary to buy food and plan meals in advance.

Finally, four items were not retained in exploratory factor analysis including price. Price was not ranked among the most important criteria when choosing dishes, whereas previous studies had demonstrated that it was an important factor in food choice [33–37]. However, price might be taken into account when purchasing foodstuff, but it is no longer important if food has already been purchased. Also, the fact that our sample comprised an overall high income/CU might explain that price was not considered a major motive in dish choices.

### Comparison of weekdays with weekends

Whatever the context (weekdays or weekends), the *healthy diet* factor ranked as most important. A potential explanation is that the French perception of cooking as a means for eating healthy is likely to be constant across the time.

As expected, *constraints* carried more weight on weekdays, whereas *pleasure* was considered more important on the weekend. In the literature, cooking has more often been considered a social event on the weekend than during weekdays [24]. Individuals are shown to cook more for pleasure on the weekend and less out of obligation [10, 45, 46]. A potential explanation to this discrepancy is the time scarcity experienced during weekdays. In line with previous studies, our results supported that less time is devoted in meal preparation during weekdays. In terms of public health strategy, promoting cooking as leisure might be an efficient lever to decrease the feeling of constraints perceived during weekdays and to promote home-food preparation.

The higher importance of *organization* during weekdays might be potentially explained by the greater importance of *constraints*, which lead some individuals to develop time-saving solutions.

For the *specific diets* factor, no difference was observed according to the context. Advice related to diets should be followed over a medium- or long-term period and is expected to be independent of the day of the week.

### Association between socio-demographic and cooking practice characteristics, and dish choice motives

Overall, our results highlighted that dish choice motives were associated with both socio-demographic characteristic and cooking practices. If cooking has been shown to be highly gendered [5, 41, 51, 52], our results suggested that gender also influence motivations when choosing the dish to prepare. Overall, women gave more importance to all motives compared with men.

The profile of individuals who gave importance to a healthy diet is consistent with trends reported in the literature: individuals more interested in healthy eating were more likely to be women, older, to have higher socio-economic status [53]. In agreement with our data, better cooking skills [54] and more time spent on meal preparation [15] were associated with healthier food choices in the literature.

Secondly, our results suggested that individuals who reported more constraints when choosing dishes had opposite socio-demographic and cooking practice characteristics compared with those who reported pleasure as important, with the exception of women who gave more importance to both factors. Women spend over twice as much time cooking as men [5]. Thus, since women are more involved in food preparation, they are more likely to consider it a household chore [24] and to experience more constraints. Parents with children reported more constraints. They had been shown in another study to consider home-cooking more as an obligation [24]. They spend more time in childcare and housework [51], and might therefore feel more time-pressured for meal preparation. Individuals with lower income reported more constraints than those with higher income. Likewise, persons with lower income have been shown to consider cooking more of an obligation, while those with higher income are more likely to obtain pleasure from food preparation [24]. Indeed, in France, the increase of food prices and the emergence of cooking trends (i.e. cooking book, cooking TV show), as well as the focus of the media on cooking have converted cooking into a leisure for only for individuals with higher income [55]. Moreover, previous studies have shown that low-income persons are more likely to experience the burden of lack of time and would thus reduce food preparation time [21, 23]. This feeling of time pressure can be explained, in particular, by constraints of lower-status jobs such as working multiple jobs, long hours, shift scheduling and overtime [56].

Among young people, lack of time is also the main barrier reported for home meal preparation [12]. In individuals aged 30 to 65, the greater importance of constraints might also be explained by the presence of children and the employment status, both of which increase time pressure. In contrast, the few constraints and high pleasure perceived by retired people might be explained by the fact that they have more time available. As expected, those who enjoyed cooking ranked constraints as less important than people who did not. Indeed, such persons have been shown to approach cooking as a leisure-time occupation and spend more time on preparing foods [24].

As regards cooking skills, those who reported having a medium level ranked constraints as more important. One potential explanation is that people with low cooking skills use more convenience foods [10, 54] and might thus place less importance on the constraints related to home meal preparation. We can hypothesize that people with high cooking skills are more likely to consider cooking as a leisurely activity, and therefore perceive less constraints.

Finally, we show that people who placed importance on *constraints* spent less time in meal preparation. Data in the literature suggest that time spent preparing foods reflects how people think about cooking. In agreement with our results, individuals who spend less than 25 min in meal preparation have been shown to approach cooking as a necessity, and therefore feel more constrained than those who spend more time preparing food and who consider cooking as more enjoyable [24]. From a public health point of view, our results suggested the importance of taking into account the perceptions of cooking, such as constraints and pleasure, in order to promote efficiently home-meal preparation. As previously suggested in the literature, developing cooking skills might be a lever to decrease the perception of constraints [57, 58] but also to increase the pleasure procured by cooking.

The *specific diet* factor included dieting for health reasons, weight loss or conviction (e.g. vegetarian, religion). This factor is therefore likely to gather individuals with very heterogeneous profiles. For example, individuals following diets for health reasons are likely to be older whereas they are less likely to follow diet to lose weight [59]. Overall, they spent more time in meal preparation which might be due to the fact that some of these diets will require a change in usual cooking practices.

Finally, people who reported importance for *organization* were more likely to have better cooking skills and to spend more time in meal preparation. Planning meal has been previously reported as a complex task when balancing the nutrition needs, food preferences and schedules of family members [60]. Therefore, having better cooking skills might be helpful to manage such practice.

### Strengths and limitations

To our knowledge, this study is the first to describe motives for dish choices. Numerous potential motives were evaluated, and differences between weekdays and weekends were assessed. Another strength of our study lies in its very large sample size and the fact that it included individuals with varying socio-demographic and lifestyle characteristics. Moreover, the use of a web-based platform enabled introducing distance between the investigator and the subject, which probably limited the social desirability bias [61]. Moreover, to conduct explanatory factor analysis, we used polychoric correlations, which are suitable for studying associations among ordered categorical variables. In line with this method, we also calculated an ordinal version of the alpha coefficient [31] and used the ULS estimation method, recommended for analyses of polychoric correlations.

Some limitations in the present study should be mentioned. First, caution is needed when generalizing our findings, as participants are recruited on a voluntary basis, and therefore are likely to be particularly health-conscious and interested in nutritional questions [62]. Only a subsample of the NutriNet-Santé cohort completed this optional questionnaire, but filling rate was similar to the one of other questionnaires completed by the cohort. Generalizing the survey to other countries is also questionable. Because of cultural differences, the importance of some dishes choice motives might vary in different countries. Urban western populations, for instance, are not restrained by food supplies or cooking equipment, whereas this might be the case in less developed countries. In contrast, time pressure has been widely described as a major issue in western societies, but not in other countries.

## Conclusion

To our knowledge, this is the first study to highlight motives underlying dish choices during home meal preparation. Based on this work, five main dimensions were identified: *healthy diet*, *constraints*, *pleasure*, *specific diets* and *organization*. Comparison of weekdays with weekends showed the *healthydiet* factor to be of greatest importance whatever the context. In turn, differences were observed for *constraints*, *pleasure* and *organization*. During weekdays, *constraints* and *organization* were ranked as more important than on weekends. On the contrary, *pleasure* was most important on weekends. Finally, dish choice motives appeared to be associated with socio-demographic and cooking practice characteristics. From a public health perspective, our findings underline the importance of understanding the context (i.e. weekdays/weekend) of dish choices, as well as socio-demographic and cooking practice characteristics of targeted individuals when designing strategies to promote home meal preparation.

## Additional file

Additional file 1:
**Dish choice questionnaire (NutriNet-Santé, 2013).** (PDF 241 kb)

## Abbreviations

BMI: Body mass index
CU: Consumption units
