# Supplementary material for: Motives for dish choices during home meal preparation: results from a large sample of the NutriNet-Santé study
Source: Int J Behav Nutr Phys Act. 2015 Sep 30;12:120. doi: 10.1186/s12966-015-0270-9 (PMC4589128; doi:10.1186/s12966-015-0270-9)
Supplement: Additional file 1: — Dish choice questionnaire (NutriNet-Santé, 2013). (PDF 241 kb) [file 12966_2015_270_MOESM1_ESM.pdf]

Additional file 1: Dish choice questionnaire (NutriNet-Santé, 2013)

Legend: This questionnaire has been adapted from the French version developed in the NutriNet-Santé study in 2013.
All use of this questionnaire should cite the following reference : Equipe de Recherche en Epidémiologie Nutritionnelle, Centre de Recherche en Epidémiologie et Statistiques, Inserm (U1153), Inra (U1125), Cnam, COMUE Sorbonne Paris Cité.

Introduction :

This questionnaire aims to better understand how you choose the dishes that you prepare in your home.
We thank you in advance for the time you may spend on this questionnaire.

Part 1: Cooking practices

In this questionnaire by "cooking" is meant the fact to prepare a cold dish (salad, fruit salad...), a hot dish, to bake, etc.

1. **Generally, in your home, who cook most often?**
   If cooking is done fairly by two people, check 2 boxes.

Weekdays	*Weekends*
☐ Myself	☐ Myself
☐ My spouse	☐ My spouse
☐ My wife	☐ My wife
☐ My mother	☐ My mother
☐ My father	☐ My father
☐ My child(ren)	☐ My child(ren)
☐ Another family member	☐ Another family member
☐ A roommate	☐ A roommate
☐ A carer	☐ A carer
☐ A neighbor, friend	☐ A neighbor, friend
☐ Other, specify:…………………………	☐ Other, specify:…………………………

2. **In general, how often do you cook <u>during the weekdays</u>?**
   - ☐ Every day twice a day or more
   - ☐ Every day once a day
   - ☐ Several times a week but not every day
   - ☐ Once a week
   - ☐ Less than once a week
   - ☐ Never

(if answer = never)

2.1 Do you usually cook on weekends ?

- ☐ Yes
- ☐ No

(if answer =no → end of the questionnaire)

3. In general, how often do you cook <u>during the weekend</u>?

- ☐ The 2 days, twice a day or more
- ☐ The 2 days, once a day
- ☐ Less than once a day
- ☐ Never

4. How much time do you usually spend on meal preparation (apart from breakfast)? *Including the cooking time*

Weekdays	***Weekend***
☐ I never cook	☐ I never cook
☐ Less than 15 minutes	☐ Less than 15 minutes
☐ Between 15 et 30 minutes	☐ Between 15 et 30 minutes
☐ Between 30 et 45 minutes	☐ Between 30 et 45 minutes
☐ Between 45 minutes et 1 hour	☐ Between 45 minutes et 1 hour
☐ More than one hour	☐ More than one hour

5. Do you enjoy cooking?

Weekdays	***Weekend***
☐ I like it very much	☐ I like it very much
☐ I like it	☐ I like it
☐ I do not like it very much	☐ I do not like it very much
☐ I do not like it at all	☐ I do not like it at all

6. Among the following statements, which one best describes your level of cooking?

- ☐ I have a very good level
- ☐ I have a good level
- ☐ I have a medium level
- ☐ I have a low level
- ☐ I cannot cook

Part 2: Dish choices

In the questionnaire, by "dish" is meant an element of a meal such as for example a cake with olives, fish en papillote, or tabbouleh. A "recipe" is the description of how a dish is prepared. For a given dish, there can be many different recipes (e.g. express tabbouleh, tabbouleh with chicken ...).

7. **In general, in your home, who mostly decide which dishes will be prepared?**
If choice is made fairly by two people, check 2 boxes.

Weekdays	***Weekend***
☐ Myself	☐ Myself
☐ My spouse	☐ My spouse
☐ My wife	☐ My wife
☐ My mother	☐ My mother
☐ My father	☐ My father
☐ My child(ren)	☐ My child(ren)
☐ Another family member	☐ Another family member
☐ A roommate	☐ A roommate
☐ A carer	☐ A carer
☐ A neighbor, friend	☐ A neighbor, friend
☐ Other, specify:............................	☐ Other, specify:............................

8. **In general, in your home, how often do you choose the dishes that will be prepared <u>during the weekdays</u>?**
☐ Always
☐ Often
☐ Sometimes
☐ Never

(if answer = never, hide the column « weekdays » in question 10 and display this question)

8.1 Do you usually choose the dishes that will be prepared during the weekend ?
☐ Yes
☐ No

(if answer =no, end of the questionnaire)

9. **In general, how often do you choose the dishes that will be prepared during the weekend?**
☐ Always
☐ Often
☐ Sometimes
☐ Never

(if answer = never, hide the column « weekend » in question 10)

10. When choosing the dishes you are going to cook, how important are the following criteria?

	Weekdays	*Weekend*
1- Time available for cooking	☐ *Very important*<br>☐ *Important*<br>☐ *Moderately important*<br>☐ *Somewhat important*<br>☐ *Not important*	☐ *Very important*<br>☐ *Important*<br>☐ *Moderately important*<br>☐ *Somewhat important*<br>☐ *Not important*
2- My cooking skills	☐ *Very important*<br>☐ *Important*<br>☐ *Moderately important*<br>☐ *Somewhat important*<br>☐ *Not important*	☐ *Very important*<br>☐ *Important*<br>☐ *Moderately important*<br>☐ *Somewhat important*<br>☐ *Not important*
3- The dish is easy to eat	☐ *Very important*<br>☐ *Important*<br>☐ *Moderately important*<br>☐ *Somewhat important*<br>☐ *Not important*	☐ *Very important*<br>☐ *Important*<br>☐ *Moderately important*<br>☐ *Somewhat important*<br>☐ *Not important*
4- Use of seasonal products	☐ *Very important*<br>☐ *Important*<br>☐ *Moderately important*<br>☐ *Somewhat important*<br>☐ *Not important*	☐ *Very important*<br>☐ *Important*<br>☐ *Moderately important*<br>☐ *Somewhat important*<br>☐ *Not important*
5- What I and/or my relatives ate during the previous days	☐ *Very important*<br>☐ *Important*<br>☐ *Moderately important*<br>☐ *Somewhat important*<br>☐ *Not important*	☐ *Very important*<br>☐ *Important*<br>☐ *Moderately important*<br>☐ *Somewhat important*<br>☐ *Not important*
6- What I planned to eat (meal planning)	☐ *Very important*<br>☐ *Important*<br>☐ *Moderately important*<br>☐ *Somewhat important*<br>☐ *Not important*	☐ *Very important*<br>☐ *Important*<br>☐ *Moderately important*<br>☐ *Somewhat important*<br>☐ *Not important*
7- The dish can be prepared beforehand	☐ *Very important*<br>☐ *Important*<br>☐ *Moderately important*<br>☐ *Somewhat important*<br>☐ *Not important*	☐ *Very important*<br>☐ *Important*<br>☐ *Moderately important*<br>☐ *Somewhat important*<br>☐ *Not important*
8- The dish can be prepared in large quantities	☐ *Very important*<br>☐ *Important*<br>☐ *Moderately important*<br>☐ *Somewhat important*<br>☐ *Not important*	☐ *Very important*<br>☐ *Important*<br>☐ *Moderately important*<br>☐ *Somewhat important*<br>☐ *Not important*
9- Number of persons eating at home	☐ *Very important*<br>☐ *Important*<br>☐ *Moderately important*<br>☐ *Somewhat important*<br>☐ *Not important*	☐ *Very important*<br>☐ *Important*<br>☐ *Moderately important*<br>☐ *Somewhat important*<br>☐ *Not important*
10- My preferences and/or those of my relatives	☐ *Very important*<br>☐ *Important*<br>☐ *Moderately important*<br>☐ *Somewhat important*<br>☐ *Not important*	☐ *Very important*<br>☐ *Important*<br>☐ *Moderately important*<br>☐ *Somewhat important*<br>☐ *Not important*
11- The dish can be adapted to please all guests	☐ *Very important*<br>☐ *Important*<br>☐ *Moderately important*<br>☐ *Somewhat important*<br>☐ *Not important*	☐ *Very important*<br>☐ *Important*<br>☐ *Moderately important*<br>☐ *Somewhat important*<br>☐ *Not important*
12- Ingredients at my disposal	☐ *Very important*<br>☐ *Important*<br>☐ *Moderately important*<br>☐ *Somewhat important*	☐ *Very important*<br>☐ *Important*<br>☐ *Moderately important*<br>☐ *Somewhat important*

	☐ *Not important*	☐ *Not important*
13- Leftovers in my refrigerator/freezer	☐ *Very important*<br>☐ *Important*<br>☐ *Moderately important*<br>☐ *Somewhat important*<br>☐ *Not important*	☐ *Very important*<br>☐ *Important*<br>☐ *Moderately important*<br>☐ *Somewhat important*<br>☐ *Not important*
14- Cooking equipment I possess	☐ *Very important*<br>☐ *Important*<br>☐ *Moderately important*<br>☐ *Somewhat important*<br>☐ *Not important*	☐ *Very important*<br>☐ *Important*<br>☐ *Moderately important*<br>☐ *Somewhat important*<br>☐ *Not important*
15- Price of ingredients	☐ *Very important*<br>☐ *Important*<br>☐ *Moderately important*<br>☐ *Somewhat important*<br>☐ *Not important*	☐ *Very important*<br>☐ *Important*<br>☐ *Moderately important*<br>☐ *Somewhat important*<br>☐ *Not important*
16- Originality of the dish	☐ *Very important*<br>☐ *Important*<br>☐ *Moderately important*<br>☐ *Somewhat important*<br>☐ *Not important*	☐ *Very important*<br>☐ *Important*<br>☐ *Moderately important*<br>☐ *Somewhat important*<br>☐ *Not important*
17- What I and/or my relatives want to eat	☐ *Very important*<br>☐ *Important*<br>☐ *Moderately important*<br>☐ *Somewhat important*<br>☐ *Not important*	☐ *Very important*<br>☐ *Important*<br>☐ *Moderately important*<br>☐ *Somewhat important*<br>☐ *Not important*
18- My eating habits and/or those of my relatives	☐ *Very important*<br>☐ *Important*<br>☐ *Moderately important*<br>☐ *Somewhat important*<br>☐ *Not important*	☐ *Very important*<br>☐ *Important*<br>☐ *Moderately important*<br>☐ *Somewhat important*<br>☐ *Not important*
19- Recipes I come across	☐ *Very important*<br>☐ *Important*<br>☐ *Moderately important*<br>☐ *Somewhat important*<br>☐ *Not important*	☐ *Very important*<br>☐ *Important*<br>☐ *Moderately important*<br>☐ *Somewhat important*<br>☐ *Not important*
20- My state of fatigue	☐ *Very important*<br>☐ *Important*<br>☐ *Moderately important*<br>☐ *Somewhat important*<br>☐ *Not important*	☐ *Very important*<br>☐ *Important*<br>☐ *Moderately important*<br>☐ *Somewhat important*<br>☐ *Not important*
21- My state of hunger and/or that of my relatives	☐ *Very important*<br>☐ *Important*<br>☐ *Moderately important*<br>☐ *Somewhat important*<br>☐ *Not important*	☐ *Very important*<br>☐ *Important*<br>☐ *Moderately important*<br>☐ *Somewhat important*<br>☐ *Not important*
22- My personal convictions and/or that of my relatives	☐ *Very important*<br>☐ *Important*<br>☐ *Moderately important*<br>☐ *Somewhat important*<br>☐ *Not important*	☐ *Very important*<br>☐ *Important*<br>☐ *Moderately important*<br>☐ *Somewhat important*<br>☐ *Not important*
23- My health status and/or those of my relatives	☐ *Very important*<br>☐ *Important*<br>☐ *Moderately important*<br>☐ *Somewhat important*<br>☐ *Not important*	☐ *Very important*<br>☐ *Important*<br>☐ *Moderately important*<br>☐ *Somewhat important*<br>☐ *Not important*
24- My eventual diet to lose weight and/or that of my relatives	☐ *Very important*<br>☐ *Important*<br>☐ *Moderately important*<br>☐ *Somewhat important*<br>☐ *Not important*	☐ *Very important*<br>☐ *Important*<br>☐ *Moderately important*<br>☐ *Somewhat important*<br>☐ *Not important*
25- The association with other dishes in terms of taste	☐ *Very important*<br>☐ *Important*<br>☐ *Moderately important*	☐ *Very important*<br>☐ *Important*<br>☐ *Moderately important*

	☐ *Somewhat important*<br>☐ *Not important*	☐ *Somewhat important*<br>☐ *Not important*
26- Nutritional balance of the meal	☐ *Very important*<br>☐ *Important*<br>☐ *Moderately important*<br>☐ *Somewhat important*<br>☐ *Not important*	☐ *Very important*<br>☐ *Important*<br>☐ *Moderately important*<br>☐ *Somewhat important*<br>☐ *Not important*
27- Nutritional balance of the dish	☐ *Very important*<br>☐ *Important*<br>☐ *Moderately important*<br>☐ *Somewhat important*<br>☐ *Not important*	☐ *Very important*<br>☐ *Important*<br>☐ *Moderately important*<br>☐ *Somewhat important*<br>☐ *Not important*

Appendix: Score computation of dish choice factors

Likert scale:

- ☐ *Very important = 5*
- ☐ *Important = 4*
- ☐ *Moderately important = 3*
- ☐ *Somewhat important = 2*
- ☐ *Not important = 1*

According to the result of the explanatory factor analysis, dish choice factors were computed as follow:

1. *Healthy diet* factor = means rating of items 4, 5, 18, 26, 27
2. *Constraints* factor = means rating of items 1, 2, 12, 13, 20, 21
3. *Pleasure* factor = means rating of items 10, 11, 16, 17, 19
4. *Specific diets* factor = means rating of items 22, 23, 24
5. *Organization* factor = means rating of items 6, 7, 8
